# Crystal structure of bis­{μ_2_-[(2-imino­cyclo­pentyl­idene)methyl­idene]aza­nido-κ^2^ *N*:*N*′}bis­[(η^5^-penta­methyl­cyclo­penta­dien­yl)zirconium(IV)] hexane monosolvate

**DOI:** 10.1107/S2056989015021234

**Published:** 2015-11-14

**Authors:** Lisanne Becker, Anke Spannenberg, Perdita Arndt, Uwe Rosenthal

**Affiliations:** a Leibniz-Institut für Katalyse e. V. an der Universität Rostock, Albert-Einstein-Strasse 29a, 18059 Rostock, Germany

**Keywords:** crystal structure, dinuclear structure, zirconocene, 1-imino-2-enimino-cyclo­pentane ligand

## Abstract

The title compound, [Zr_2_(C_10_H_15_)_4_(C_6_H_6_N_2_)_2_]·C_6_H_14_, was obtained by the stoichiometric reaction of adipo­nitrile with [Zr(C_10_H_15_)_2_(η^2^-Me_3_SiC_2_SiMe_3_)]. Intra­molecular nitrile–nitrile couplings and deprotonation of the substrate produced the (1-imino-2-enimino)­cyclo­pentane ligand, which functions as a five-membered bridge between the two metal atoms. The Zr^IV^ atom exhibits a distorted tetra­hedral coordination sphere defined by two penta­methyl­cyclo­penta­dienyl ligands, by the imino unit of one (1-imino-2-enimino)­cyclo­pentane and by the enimino unit of the second (1-imino-2-enimino)­cyclo­pentane. The cyclo­pentane ring of the ligand shows an envelope conformation. The asymmetric unit contains one half of the complex and one half of the hexane solvent mol­ecule, both being completed by the application of inversion symmetry. One of the penta­methyl­cyclo­penta­dienyl ligands is disordered over two sets of sites with a refined occupancy ratio of 0.8111 (3):0.189 (3). In the crystal, the complex mol­ecules are packed into rods extending along [100], with the solvent mol­ecules located in between. The rods are arranged in a distorted hexa­gonal packing.

## Related literature   

For more information about group 4 metallocene chemistry with di­cyano compounds, see: Becker, Arndt *et al.* (2015 ▸). For group 4 complexes with comparable five-membered en–dimine ligands, see: Becker, Haehnel *et al.* (2015 ▸). For intra­molecular C—C coupling reactions of adipo­nitrile, see: Thorpe (1909 ▸); Schroeder & Rigby (1949 ▸).
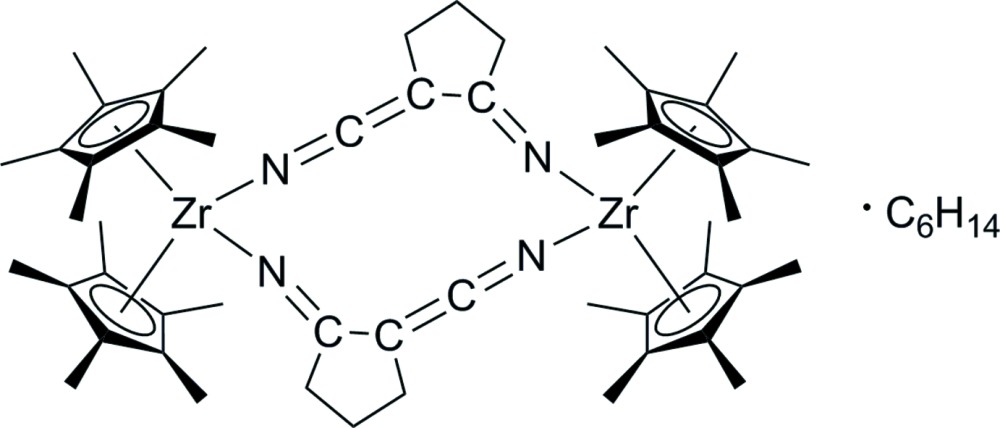


## Experimental   

### Crystal data  

[Zr_2_(C_10_H_15_)_4_(C_6_H_6_N_2_)_2_]·C_6_H_14_
*M* *_r_* = 1021.74
Monoclinic, 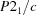

*a* = 13.4862 (8) Å
*b* = 16.9048 (11) Å
*c* = 13.0151 (8) Å
β = 117.7232 (15)°
*V* = 2626.6 (3) Å^3^
*Z* = 2
Mo *K*α radiation
μ = 0.44 mm^−1^
*T* = 150 K
0.35 × 0.27 × 0.18 mm

### Data collection  

Bruker APEXII CCD diffractometer
Absorption correction: multi-scan (*SADABS*; Bruker, 2014 ▸) *T*_min_ = 0.88, *T*_max_ = 0.93
60472 measured reflections
6341 independent reflections
5621 reflections with *I* > 2σ(*I*)
*R*_int_ = 0.028

### Refinement  

*R*[*F* ^2^ > 2σ(*F* ^2^)] = 0.035
*wR*(*F* ^2^) = 0.094
*S* = 1.06
6341 reflections
386 parameters
239 restraints
H-atom parameters constrained
Δρ_max_ = 0.88 e Å^−3^
Δρ_min_ = −0.43 e Å^−3^

### 

Data collection: *APEX2* (Bruker, 2014 ▸); cell refinement: *SAINT* (Bruker, 2014 ▸); data reduction: *SAINT*; program(s) used to solve structure: *SHELXS97* (Sheldrick, 2008 ▸); program(s) used to refine structure: *SHELXL2014* (Sheldrick, 2015 ▸); molecular graphics: *XP* in *SHELXTL* (Sheldrick, 2008 ▸) and *Mercury* (Macrae *et al.*, 2006 ▸); software used to prepare material for publication: *publCIF* (Westrip, 2010 ▸).

## Supplementary Material

Crystal structure: contains datablock(s) I, New_Global_Publ_Block. DOI: 10.1107/S2056989015021234/wm5234sup1.cif

**Fig. 1.**
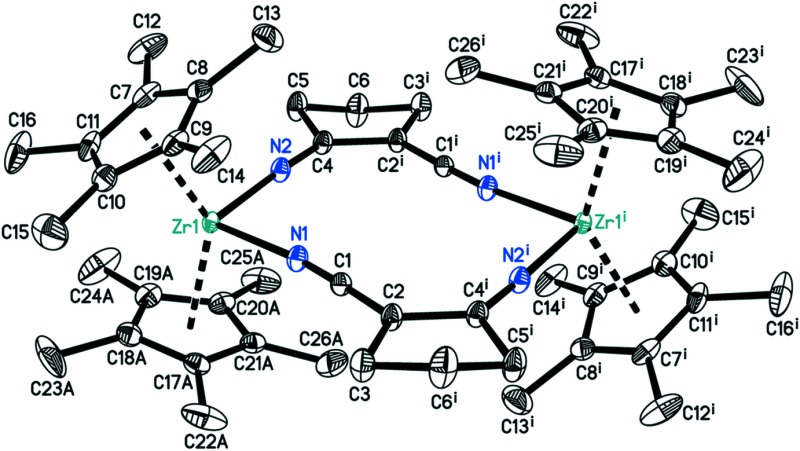
The molecular structure of the title compound. The minor occupied atoms of the disordered pentamethylcyclopentadienyl ligands, hydrogen atoms and the solvent molecule are omitted for clarity. Displacement ellipsoids are drawn at the 30% probability level. [Symmetry code: (i) -x + 2, -y + 1, -z + 2.]

Structure factors: contains datablock(s) I. DOI: 10.1107/S2056989015021234/wm5234Isup2.hkl

x y z . DOI: 10.1107/S2056989015021234/wm5234fig1.tif
The mol­ecular structure of the title compound. The minor occupied atoms of the disordered penta­methyl­cyclo­penta­dienyl ligands, hydrogen atoms and the solvent mol­ecule are omitted for clarity. Displacement ellipsoids are drawn at the 30% probability level. [Symmetry code: (i) −*x* + 2, −*y* + 1, −*z* + 2.]

**Fig. 2.**
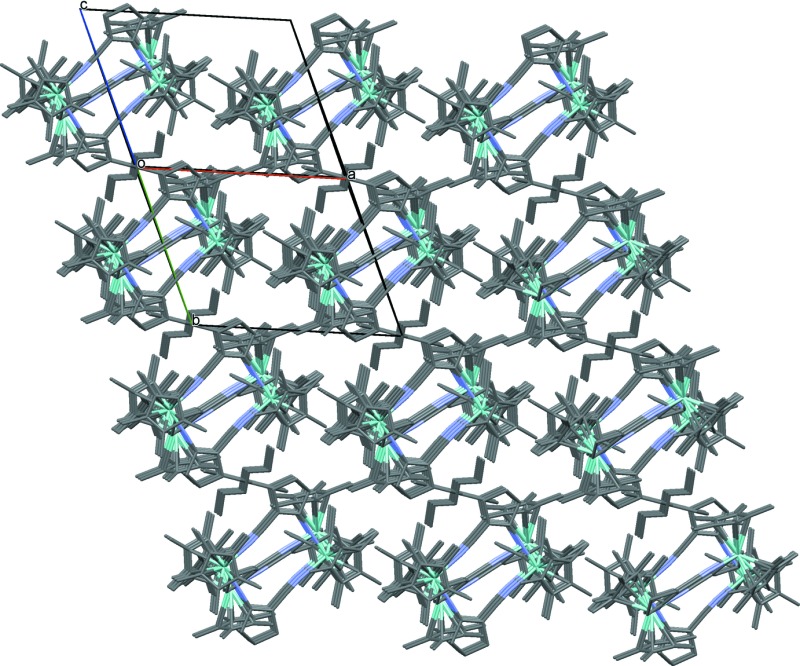
Crystal packing of the title compound (capped sticks) in a projection along [011].

. DOI: 10.1107/S2056989015021234/wm5234fig2.tif
Crystal packing of the title compound (capped sticks) in a projection along [011].

CCDC reference: 1435859

Additional supporting information:  crystallographic information; 3D view; checkCIF report

## supplementary crystallographic information

### S1. Synthesis and crystallization

To a solution of [Zr(C_10_H_15_)_2_(η^2^-Me_3_SiC_2_SiMe_3_)] (0.266 g, 0.5 mmol) in 10 ml of toluene, adipo­nitrile (0.056 ml, 0.5 mmol) was added dropwise under stirring. The color changed to yellow and during 24 h to orange. All volatiles were removed *in vacuo* and the orange residue extracted with *n*-hexane. Red crystals formed within three weeks at ambient temperature. MS: *m/z* (EI): 932 (6) [M]^+^, 799 (4) [*M*-Cp*]^+^, 360 (4) [Cp*_2_Zr]^+^, 135 (15) [Cp*]^+^.

### S2. Refinement

H atoms were placed in idealized positions with *d*(C—H) = 0.99 Å (CH_2_), 0.98 Å (CH_3_) and refined using a riding model with *U*_iso_(H) fixed at 1.2*U*_eq_(C) for CH_2_ and 1.5*U*_eq_(C) for CH_3_. A rotating model was used for fully occupied methyl groups. One cyclo­penta­dienyl ligand is disordered over two sets of sites with refined occupacies of 0.8111 (3):0.189 (3). DANG and SAME instructions were used to improve the geometry of the penta­methyl­cyclo­penta­dienyl ring C17B–C26B. Additionally, anisotropic displacement parameters of atoms C17A–C21A, C17B–C21B and C22A–C26A, C22B–C26B were restrained to be equal (SIMU), respectively. The maximum remaining electron density in the final difference Fourier map is located 0.77 Å from Zr1 and the minimum electron density 0.39 Å from C24B.

### Crystal data

**Table:**

[Zr_2_(C_10_H_15_)_4_(C_6_H_6_N_2_)_2_]·C_6_H_14_	*F*(000) = 1084
*M**_r_* = 1021.74	*D*_x_ = 1.292 Mg m^−^^3^
Monoclinic, *P*2_1_/*c*	Mo *K*α radiation, λ = 0.71073 Å
*a* = 13.4862 (8) Å	Cell parameters from 9621 reflections
*b* = 16.9048 (11) Å	θ = 3.0–28.8°
*c* = 13.0151 (8) Å	µ = 0.44 mm^−^^1^
β = 117.7232 (15)°	*T* = 150 K
*V* = 2626.6 (3) Å^3^	Prism, red
*Z* = 2	0.35 × 0.27 × 0.18 mm

### Data collection

**Table:**

Bruker APEXII CCD diffractometer	6341 independent reflections
Radiation source: fine-focus sealed tube	5621 reflections with *I* > 2σ(*I*)
Detector resolution: 8.3333 pixels mm^-1^	*R*_int_ = 0.028
φ and ω scans	θ_max_ = 28.0°, θ_min_ = 1.7°
Absorption correction: multi-scan (*SADABS*; Bruker, 2014)	*h* = −17→17
*T*_min_ = 0.88, *T*_max_ = 0.93	*k* = −22→22
60472 measured reflections	*l* = −16→17

### Refinement

**Table:**

Refinement on *F*^2^	239 restraints
Least-squares matrix: full	Hydrogen site location: inferred from neighbouring sites
*R*[*F*^2^ > 2σ(*F*^2^)] = 0.035	H-atom parameters constrained
*wR*(*F*^2^) = 0.094	*w* = 1/[σ^2^(*F*_o_^2^) + (0.0437*P*)^2^ + 3.0941*P*] where *P* = (*F*_o_^2^ + 2*F*_c_^2^)/3
*S* = 1.06	(Δ/σ)_max_ = 0.001
6341 reflections	Δρ_max_ = 0.88 e Å^−^^3^
386 parameters	Δρ_min_ = −0.43 e Å^−^^3^

### Special details

**Table:**

Geometry. All e.s.d.'s (except the e.s.d. in the dihedral angle between two l.s. planes) are estimated using the full covariance matrix. The cell e.s.d.'s are taken into account individually in the estimation of e.s.d.'s in distances, angles and torsion angles; correlations between e.s.d.'s in cell parameters are only used when they are defined by crystal symmetry. An approximate (isotropic) treatment of cell e.s.d.'s is used for estimating e.s.d.'s involving l.s. planes.

### Fractional atomic coordinates and isotropic or equivalent isotropic displacement parameters (Å^2^)

**Table:**

	*x*	*y*	*z*	*U*_iso_*/*U*_eq_	Occ. (<1)
Zr1	0.74138 (2)	0.57267 (2)	0.87471 (2)	0.01961 (7)	
N1	0.91400 (14)	0.59393 (11)	1.01612 (16)	0.0266 (4)	
N2	0.78112 (14)	0.48191 (10)	0.80846 (15)	0.0269 (4)	
C1	0.99978 (17)	0.60769 (12)	1.09499 (18)	0.0236 (4)	
C2	1.09941 (17)	0.62794 (13)	1.18946 (18)	0.0269 (4)	
C3	1.1146 (2)	0.70152 (14)	1.2619 (2)	0.0370 (5)	
H3A	1.0825	0.6943	1.3160	0.044*	
H3B	1.0796	0.7483	1.2123	0.044*	
C4	0.80110 (18)	0.41727 (12)	0.76840 (19)	0.0268 (4)	
C5	0.7144 (2)	0.37526 (15)	0.6595 (2)	0.0385 (6)	
H5A	0.6395	0.3766	0.6558	0.046*	
H5B	0.7098	0.4002	0.5885	0.046*	
C6	0.7577 (2)	0.29049 (16)	0.6724 (3)	0.0472 (7)	
H6A	0.7337	0.2658	0.5955	0.057*	
H6B	0.7300	0.2580	0.7170	0.057*	
C7	0.6755 (2)	0.63050 (14)	0.67304 (19)	0.0354 (5)	
C8	0.7897 (2)	0.64930 (14)	0.7346 (2)	0.0327 (5)	
C9	0.80379 (18)	0.70401 (13)	0.82182 (19)	0.0308 (5)	
C10	0.6973 (2)	0.71903 (13)	0.8136 (2)	0.0346 (5)	
C11	0.61826 (19)	0.67272 (14)	0.7208 (2)	0.0367 (5)	
C12	0.6217 (3)	0.57888 (18)	0.5670 (2)	0.0609 (9)	
H12A	0.5653	0.5450	0.5724	0.091*	
H12B	0.6790	0.5458	0.5620	0.091*	
H12C	0.5857	0.6122	0.4976	0.091*	
C13	0.8791 (3)	0.6204 (2)	0.7062 (3)	0.0585 (8)	
H13A	0.8664	0.5643	0.6846	0.088*	
H13B	0.9526	0.6268	0.7741	0.088*	
H13C	0.8767	0.6511	0.6412	0.088*	
C14	0.9082 (2)	0.74771 (18)	0.9002 (3)	0.0543 (8)	
H14A	0.9737	0.7161	0.9124	0.081*	
H14B	0.9092	0.7573	0.9750	0.081*	
H14C	0.9101	0.7984	0.8647	0.081*	
C15	0.6731 (3)	0.78178 (17)	0.8802 (3)	0.0634 (9)	
H15A	0.6882	0.8340	0.8577	0.095*	
H15B	0.7209	0.7737	0.9635	0.095*	
H15C	0.5942	0.7787	0.8628	0.095*	
C16	0.4921 (2)	0.6800 (2)	0.6629 (3)	0.0680 (11)	
H16A	0.4666	0.7106	0.5911	0.102*	
H16B	0.4695	0.7070	0.7153	0.102*	
H16C	0.4585	0.6271	0.6448	0.102*	
C17A	0.7126 (3)	0.56342 (19)	1.0548 (3)	0.0319 (5)	0.811 (3)
C18A	0.6044 (3)	0.58128 (18)	0.9616 (3)	0.0337 (5)	0.811 (3)
C19A	0.5710 (3)	0.51561 (19)	0.8842 (3)	0.0351 (5)	0.811 (3)
C20A	0.6543 (3)	0.45762 (18)	0.9317 (3)	0.0334 (5)	0.811 (3)
C21A	0.7415 (2)	0.48706 (18)	1.0345 (2)	0.0311 (5)	0.811 (3)
C22A	0.7769 (4)	0.6134 (3)	1.1598 (4)	0.0651 (11)	0.811 (3)
H22A	0.7379	0.6637	1.1511	0.098*	0.811 (3)
H22B	0.7831	0.5857	1.2287	0.098*	0.811 (3)
H22C	0.8520	0.6234	1.1685	0.098*	0.811 (3)
C23A	0.5301 (5)	0.6474 (3)	0.9602 (6)	0.091 (2)	0.811 (3)
H23A	0.5736	0.6849	1.0224	0.137*	0.811 (3)
H23B	0.4993	0.6746	0.8851	0.137*	0.811 (3)
H23C	0.4687	0.6260	0.9722	0.137*	0.811 (3)
C24A	0.4575 (3)	0.5009 (4)	0.7824 (4)	0.085 (2)	0.811 (3)
H24A	0.4129	0.5495	0.7639	0.127*	0.811 (3)
H24B	0.4667	0.4847	0.7151	0.127*	0.811 (3)
H24C	0.4192	0.4589	0.8021	0.127*	0.811 (3)
C25A	0.6508 (4)	0.3744 (2)	0.8886 (4)	0.0628 (11)	0.811 (3)
H25A	0.7210	0.3473	0.9392	0.094*	0.811 (3)
H25B	0.5879	0.3458	0.8894	0.094*	0.811 (3)
H25C	0.6411	0.3760	0.8092	0.094*	0.811 (3)
C26A	0.8461 (4)	0.4421 (3)	1.1119 (4)	0.0615 (10)	0.811 (3)
H26A	0.8943	0.4752	1.1783	0.092*	0.811 (3)
H26B	0.8260	0.3939	1.1397	0.092*	0.811 (3)
H26C	0.8862	0.4279	1.0681	0.092*	0.811 (3)
C17B	0.6671 (9)	0.5907 (6)	1.0239 (9)	0.0311 (8)	0.189 (3)
C18B	0.5724 (9)	0.5715 (6)	0.9191 (10)	0.0315 (8)	0.189 (3)
C19B	0.5851 (10)	0.4934 (6)	0.8913 (12)	0.0327 (8)	0.189 (3)
C20B	0.6885 (9)	0.4642 (6)	0.9776 (9)	0.0333 (7)	0.189 (3)
C21B	0.7376 (9)	0.5240 (6)	1.0608 (10)	0.0326 (7)	0.189 (3)
C22B	0.6830 (13)	0.6651 (7)	1.0924 (12)	0.072 (3)	0.189 (3)
H22D	0.6225	0.7023	1.0473	0.109*	0.189 (3)
H22E	0.6817	0.6526	1.1653	0.109*	0.189 (3)
H22F	0.7552	0.6890	1.1094	0.109*	0.189 (3)
C23B	0.4652 (10)	0.6185 (9)	0.8646 (14)	0.093 (3)	0.189 (3)
H23D	0.4786	0.6714	0.8992	0.139*	0.189 (3)
H23E	0.4392	0.6231	0.7809	0.139*	0.189 (3)
H23F	0.4080	0.5916	0.8781	0.139*	0.189 (3)
C24B	0.5026 (12)	0.4475 (8)	0.7885 (12)	0.088 (3)	0.189 (3)
H24D	0.4377	0.4809	0.7417	0.133*	0.189 (3)
H24E	0.5382	0.4307	0.7413	0.133*	0.189 (3)
H24F	0.4781	0.4008	0.8152	0.133*	0.189 (3)
C25B	0.7292 (13)	0.3811 (6)	0.9867 (13)	0.067 (2)	0.189 (3)
H25D	0.8033	0.3763	1.0542	0.100*	0.189 (3)
H25E	0.6768	0.3452	0.9957	0.100*	0.189 (3)
H25F	0.7343	0.3673	0.9162	0.100*	0.189 (3)
C26B	0.8391 (10)	0.5150 (9)	1.1768 (9)	0.068 (2)	0.189 (3)
H26D	0.8735	0.4632	1.1809	0.102*	0.189 (3)
H26E	0.8931	0.5568	1.1868	0.102*	0.189 (3)
H26F	0.8169	0.5189	1.2384	0.102*	0.189 (3)
C27	0.8172 (3)	0.1359 (2)	0.9157 (3)	0.0627 (9)	
H27A	0.8707	0.1794	0.9492	0.094*	
H27B	0.7497	0.1473	0.9234	0.094*	
H27C	0.7971	0.1300	0.8334	0.094*	
C28	0.8691 (3)	0.0609 (2)	0.9785 (3)	0.0600 (8)	
H28A	0.8135	0.0177	0.9462	0.072*	
H28B	0.8884	0.0671	1.0614	0.072*	
C29	0.9735 (3)	0.0376 (2)	0.9703 (3)	0.0538 (7)	
H29A	0.9542	0.0328	0.8872	0.065*	
H29B	1.0292	0.0807	1.0037	0.065*	

### Atomic displacement parameters (Å^2^)

**Table:**

	*U*^11^	*U*^22^	*U*^33^	*U*^12^	*U*^13^	*U*^23^
Zr1	0.01503 (10)	0.01940 (10)	0.02077 (10)	−0.00053 (6)	0.00526 (7)	0.00055 (7)
N1	0.0203 (8)	0.0255 (8)	0.0282 (9)	−0.0002 (7)	0.0063 (7)	−0.0009 (7)
N2	0.0216 (8)	0.0258 (8)	0.0264 (9)	0.0013 (7)	0.0054 (7)	−0.0023 (7)
C1	0.0228 (10)	0.0220 (9)	0.0252 (9)	0.0009 (7)	0.0105 (8)	−0.0002 (8)
C2	0.0206 (9)	0.0270 (10)	0.0256 (10)	0.0014 (8)	0.0045 (8)	−0.0057 (8)
C3	0.0300 (11)	0.0338 (12)	0.0358 (12)	0.0050 (9)	0.0056 (10)	−0.0119 (10)
C4	0.0216 (10)	0.0265 (10)	0.0249 (10)	0.0000 (8)	0.0046 (8)	−0.0032 (8)
C5	0.0252 (11)	0.0395 (13)	0.0328 (12)	0.0039 (9)	−0.0017 (9)	−0.0122 (10)
C6	0.0344 (13)	0.0370 (13)	0.0458 (15)	0.0015 (10)	−0.0019 (11)	−0.0184 (11)
C7	0.0397 (13)	0.0300 (11)	0.0233 (10)	−0.0055 (9)	0.0035 (9)	0.0065 (9)
C8	0.0348 (12)	0.0337 (11)	0.0322 (11)	0.0020 (9)	0.0178 (10)	0.0098 (9)
C9	0.0273 (11)	0.0285 (10)	0.0289 (10)	−0.0077 (8)	0.0067 (9)	0.0076 (8)
C10	0.0429 (13)	0.0220 (10)	0.0417 (13)	0.0045 (9)	0.0221 (11)	0.0081 (9)
C11	0.0239 (11)	0.0331 (12)	0.0425 (13)	0.0020 (9)	0.0066 (10)	0.0174 (10)
C12	0.084 (2)	0.0494 (17)	0.0263 (13)	−0.0163 (16)	0.0060 (14)	0.0002 (11)
C13	0.063 (2)	0.0630 (19)	0.072 (2)	0.0142 (16)	0.0507 (18)	0.0194 (16)
C14	0.0448 (15)	0.0492 (16)	0.0435 (15)	−0.0241 (13)	−0.0010 (12)	0.0156 (12)
C15	0.099 (3)	0.0283 (13)	0.086 (2)	0.0079 (15)	0.062 (2)	0.0024 (14)
C16	0.0274 (14)	0.066 (2)	0.088 (3)	0.0063 (13)	0.0080 (15)	0.0405 (19)
C17A	0.0322 (10)	0.0368 (10)	0.0330 (9)	−0.0056 (8)	0.0206 (8)	−0.0031 (8)
C18A	0.0305 (10)	0.0373 (10)	0.0390 (10)	−0.0041 (8)	0.0210 (8)	0.0020 (9)
C19A	0.0307 (10)	0.0371 (11)	0.0371 (10)	−0.0101 (9)	0.0154 (8)	0.0053 (10)
C20A	0.0357 (10)	0.0337 (10)	0.0315 (10)	−0.0093 (8)	0.0164 (8)	0.0029 (9)
C21A	0.0340 (10)	0.0346 (10)	0.0287 (9)	−0.0043 (8)	0.0181 (8)	0.0020 (8)
C22A	0.075 (2)	0.085 (3)	0.056 (2)	−0.034 (2)	0.0481 (19)	−0.0336 (19)
C23A	0.105 (4)	0.069 (3)	0.167 (6)	0.039 (3)	0.120 (5)	0.043 (3)
C24A	0.037 (2)	0.123 (5)	0.061 (2)	−0.043 (3)	−0.0060 (19)	0.038 (3)
C25A	0.105 (3)	0.0362 (17)	0.069 (2)	−0.0208 (19)	0.059 (2)	−0.0029 (16)
C26A	0.067 (2)	0.071 (2)	0.0519 (19)	0.0103 (18)	0.0324 (17)	0.0281 (17)
C17B	0.0321 (12)	0.0355 (12)	0.0320 (12)	−0.0052 (11)	0.0202 (11)	0.0008 (11)
C18B	0.0303 (12)	0.0366 (12)	0.0336 (12)	−0.0058 (11)	0.0200 (11)	0.0008 (11)
C19B	0.0312 (12)	0.0354 (12)	0.0351 (12)	−0.0079 (11)	0.0185 (11)	0.0020 (12)
C20B	0.0329 (11)	0.0351 (11)	0.0339 (11)	−0.0072 (10)	0.0173 (10)	0.0023 (11)
C21B	0.0330 (11)	0.0354 (12)	0.0329 (11)	−0.0060 (10)	0.0182 (10)	0.0018 (11)
C22B	0.086 (4)	0.087 (5)	0.059 (4)	−0.035 (4)	0.046 (4)	−0.031 (4)
C23B	0.105 (6)	0.075 (5)	0.165 (7)	0.041 (5)	0.119 (6)	0.043 (5)
C24B	0.044 (4)	0.127 (6)	0.063 (4)	−0.043 (4)	−0.002 (4)	0.033 (5)
C25B	0.089 (3)	0.054 (3)	0.066 (3)	−0.003 (3)	0.045 (3)	0.013 (3)
C26B	0.073 (3)	0.082 (3)	0.057 (3)	−0.011 (3)	0.036 (3)	0.002 (3)
C27	0.0449 (17)	0.088 (3)	0.0529 (18)	−0.0055 (16)	0.0207 (14)	0.0056 (17)
C28	0.0496 (18)	0.081 (2)	0.0554 (19)	−0.0051 (16)	0.0295 (15)	0.0038 (17)
C29	0.0528 (17)	0.069 (2)	0.0460 (16)	−0.0117 (15)	0.0282 (14)	−0.0030 (14)

### Geometric parameters (Å, º)

**Table:**

Zr1—N2	1.9532 (18)	C18A—C23A	1.496 (5)
Zr1—N1	2.2248 (17)	C19A—C20A	1.400 (4)
Zr1—C21A	2.533 (3)	C19A—C24A	1.507 (4)
Zr1—C7	2.543 (2)	C20A—C21A	1.399 (4)
Zr1—C19A	2.548 (4)	C20A—C25A	1.507 (5)
Zr1—C17A	2.552 (3)	C21A—C26A	1.503 (5)
Zr1—C20A	2.554 (3)	C22A—H22A	0.9800
Zr1—C8	2.556 (2)	C22A—H22B	0.9800
Zr1—C11	2.556 (2)	C22A—H22C	0.9800
Zr1—C20B	2.560 (13)	C23A—H23A	0.9800
Zr1—C9	2.579 (2)	C23A—H23B	0.9800
Zr1—C21B	2.581 (12)	C23A—H23C	0.9800
N1—C1	1.158 (3)	C24A—H24A	0.9800
N2—C4	1.292 (3)	C24A—H24B	0.9800
C1—C2	1.377 (3)	C24A—H24C	0.9800
C2—C4^i^	1.414 (3)	C25A—H25A	0.9800
C2—C3	1.516 (3)	C25A—H25B	0.9800
C3—C6^i^	1.531 (3)	C25A—H25C	0.9800
C3—H3A	0.9900	C26A—H26A	0.9800
C3—H3B	0.9900	C26A—H26B	0.9800
C4—C2^i^	1.414 (3)	C26A—H26C	0.9800
C4—C5	1.529 (3)	C17B—C21B	1.407 (11)
C5—C6	1.527 (3)	C17B—C18B	1.405 (11)
C5—H5A	0.9900	C17B—C22B	1.499 (11)
C5—H5B	0.9900	C18B—C19B	1.399 (11)
C6—C3^i^	1.531 (3)	C18B—C23B	1.508 (11)
C6—H6A	0.9900	C19B—C20B	1.412 (11)
C6—H6B	0.9900	C19B—C24B	1.499 (11)
C7—C11	1.392 (4)	C20B—C21B	1.402 (11)
C7—C8	1.402 (3)	C20B—C25B	1.494 (11)
C7—C12	1.503 (4)	C21B—C26B	1.501 (11)
C8—C9	1.407 (3)	C22B—H22D	0.9800
C8—C13	1.498 (4)	C22B—H22E	0.9800
C9—C10	1.413 (3)	C22B—H22F	0.9800
C9—C14	1.494 (3)	C23B—H23D	0.9800
C10—C11	1.418 (4)	C23B—H23E	0.9800
C10—C15	1.498 (4)	C23B—H23F	0.9800
C11—C16	1.511 (3)	C24B—H24D	0.9800
C12—H12A	0.9800	C24B—H24E	0.9800
C12—H12B	0.9800	C24B—H24F	0.9800
C12—H12C	0.9800	C25B—H25D	0.9800
C13—H13A	0.9800	C25B—H25E	0.9800
C13—H13B	0.9800	C25B—H25F	0.9800
C13—H13C	0.9800	C26B—H26D	0.9800
C14—H14A	0.9800	C26B—H26E	0.9800
C14—H14B	0.9800	C26B—H26F	0.9800
C14—H14C	0.9800	C27—C28	1.495 (5)
C15—H15A	0.9800	C27—H27A	0.9800
C15—H15B	0.9800	C27—H27B	0.9800
C15—H15C	0.9800	C27—H27C	0.9800
C16—H16A	0.9800	C28—C29	1.513 (4)
C16—H16B	0.9800	C28—H28A	0.9900
C16—H16C	0.9800	C28—H28B	0.9900
C17A—C21A	1.408 (4)	C29—C29^ii^	1.486 (7)
C17A—C18A	1.430 (5)	C29—H29A	0.9900
C17A—C22A	1.494 (5)	C29—H29B	0.9900
C18A—C19A	1.424 (5)		
			
N2—Zr1—N1	95.49 (7)	C11—C16—H16C	109.5
N2—Zr1—C21A	91.04 (9)	H16A—C16—H16C	109.5
N1—Zr1—C21A	79.18 (8)	H16B—C16—H16C	109.5
N2—Zr1—C7	83.23 (8)	C21A—C17A—C18A	107.3 (3)
N1—Zr1—C7	121.36 (7)	C21A—C17A—C22A	126.6 (3)
C21A—Zr1—C7	159.03 (9)	C18A—C17A—C22A	125.9 (3)
N2—Zr1—C19A	99.38 (10)	C21A—C17A—Zr1	73.18 (16)
N1—Zr1—C19A	129.90 (9)	C18A—C17A—Zr1	74.91 (17)
C21A—Zr1—C19A	53.14 (10)	C22A—C17A—Zr1	122.3 (2)
C7—Zr1—C19A	107.80 (10)	C19A—C18A—C17A	107.3 (3)
N2—Zr1—C17A	123.19 (9)	C19A—C18A—C23A	125.7 (4)
N1—Zr1—C17A	78.34 (9)	C17A—C18A—C23A	125.6 (4)
C21A—Zr1—C17A	32.16 (10)	C19A—C18A—Zr1	72.6 (2)
C7—Zr1—C17A	147.37 (10)	C17A—C18A—Zr1	72.74 (17)
C19A—Zr1—C17A	53.59 (10)	C23A—C18A—Zr1	130.5 (2)
N2—Zr1—C20A	77.76 (9)	C20A—C19A—C18A	108.0 (3)
N1—Zr1—C20A	109.17 (9)	C20A—C19A—C24A	123.4 (4)
C21A—Zr1—C20A	31.92 (9)	C18A—C19A—C24A	127.5 (4)
C7—Zr1—C20A	127.27 (9)	C20A—C19A—Zr1	74.33 (19)
C19A—Zr1—C20A	31.84 (10)	C18A—C19A—Zr1	75.12 (18)
C17A—Zr1—C20A	53.10 (10)	C24A—C19A—Zr1	126.1 (3)
N2—Zr1—C8	82.51 (8)	C19A—C20A—C21A	108.6 (3)
N1—Zr1—C8	89.57 (7)	C19A—C20A—C25A	127.6 (3)
C21A—Zr1—C8	166.47 (9)	C21A—C20A—C25A	123.7 (3)
C7—Zr1—C8	31.92 (8)	C19A—C20A—Zr1	73.83 (19)
C19A—Zr1—C8	139.59 (9)	C21A—C20A—Zr1	73.19 (16)
C17A—Zr1—C8	152.17 (9)	C25A—C20A—Zr1	122.8 (2)
C20A—Zr1—C8	153.83 (9)	C20A—C21A—C17A	108.8 (3)
N2—Zr1—C11	112.67 (8)	C20A—C21A—C26A	124.8 (3)
N1—Zr1—C11	126.12 (7)	C17A—C21A—C26A	126.4 (3)
C21A—Zr1—C11	140.67 (9)	C20A—C21A—Zr1	74.88 (16)
C7—Zr1—C11	31.69 (9)	C17A—C21A—Zr1	74.66 (16)
C19A—Zr1—C11	90.96 (9)	C26A—C21A—Zr1	118.8 (2)
C17A—Zr1—C11	116.05 (10)	C17A—C22A—H22A	109.5
C20A—Zr1—C11	120.83 (9)	C17A—C22A—H22B	109.5
C8—Zr1—C11	52.69 (8)	H22A—C22A—H22B	109.5
N2—Zr1—C20B	82.5 (3)	C17A—C22A—H22C	109.5
N1—Zr1—C20B	97.0 (2)	H22A—C22A—H22C	109.5
C7—Zr1—C20B	140.1 (2)	H22B—C22A—H22C	109.5
C8—Zr1—C20B	164.1 (3)	C18A—C23A—H23A	109.5
C11—Zr1—C20B	130.3 (2)	C18A—C23A—H23B	109.5
N2—Zr1—C9	111.58 (8)	H23A—C23A—H23B	109.5
N1—Zr1—C9	74.68 (7)	C18A—C23A—H23C	109.5
C21A—Zr1—C9	146.65 (9)	H23A—C23A—H23C	109.5
C7—Zr1—C9	52.71 (7)	H23B—C23A—H23C	109.5
C19A—Zr1—C9	138.82 (9)	C19A—C24A—H24A	109.5
C17A—Zr1—C9	120.38 (9)	C19A—C24A—H24B	109.5
C20A—Zr1—C9	169.86 (9)	H24A—C24A—H24B	109.5
C8—Zr1—C9	31.80 (8)	C19A—C24A—H24C	109.5
C11—Zr1—C9	52.77 (7)	H24A—C24A—H24C	109.5
C20B—Zr1—C9	164.0 (2)	H24B—C24A—H24C	109.5
N2—Zr1—C21B	107.1 (2)	C20A—C25A—H25A	109.5
N1—Zr1—C21B	75.2 (2)	C20A—C25A—H25B	109.5
C7—Zr1—C21B	160.3 (3)	H25A—C25A—H25B	109.5
C8—Zr1—C21B	162.5 (2)	C20A—C25A—H25C	109.5
C11—Zr1—C21B	131.1 (2)	H25A—C25A—H25C	109.5
C20B—Zr1—C21B	31.6 (3)	H25B—C25A—H25C	109.5
C9—Zr1—C21B	132.5 (2)	C21A—C26A—H26A	109.5
C1—N1—Zr1	174.30 (18)	C21A—C26A—H26B	109.5
C4—N2—Zr1	173.79 (17)	H26A—C26A—H26B	109.5
N1—C1—C2	176.8 (2)	C21A—C26A—H26C	109.5
C1—C2—C4^i^	124.56 (19)	H26A—C26A—H26C	109.5
C1—C2—C3	123.53 (19)	H26B—C26A—H26C	109.5
C4^i^—C2—C3	111.91 (18)	C21B—C17B—C18B	108.1 (9)
C2—C3—C6^i^	102.16 (19)	C21B—C17B—C22B	125.6 (8)
C2—C3—H3A	111.3	C18B—C17B—C22B	125.9 (8)
C6^i^—C3—H3A	111.3	C21B—C17B—Zr1	74.1 (7)
C2—C3—H3B	111.3	C18B—C17B—Zr1	74.8 (7)
C6^i^—C3—H3B	111.3	C22B—C17B—Zr1	123.2 (10)
H3A—C3—H3B	109.2	C19B—C18B—C17B	107.7 (10)
N2—C4—C2^i^	129.70 (19)	C19B—C18B—C23B	125.5 (8)
N2—C4—C5	123.64 (19)	C17B—C18B—C23B	125.0 (8)
C2^i^—C4—C5	106.65 (18)	C19B—C18B—Zr1	74.0 (8)
C6—C5—C4	104.20 (18)	C17B—C18B—Zr1	73.7 (7)
C6—C5—H5A	110.9	C23B—C18B—Zr1	129.7 (10)
C4—C5—H5A	110.9	C20B—C19B—C18B	108.5 (10)
C6—C5—H5B	110.9	C20B—C19B—C24B	125.3 (8)
C4—C5—H5B	110.9	C18B—C19B—C24B	126.2 (8)
H5A—C5—H5B	108.9	C20B—C19B—Zr1	72.9 (8)
C5—C6—C3^i^	104.6 (2)	C18B—C19B—Zr1	74.7 (8)
C5—C6—H6A	110.8	C24B—C19B—Zr1	119.2 (12)
C3^i^—C6—H6A	110.8	C19B—C20B—C21B	107.4 (9)
C5—C6—H6B	110.8	C19B—C20B—C25B	125.6 (8)
C3^i^—C6—H6B	110.8	C21B—C20B—C25B	126.4 (8)
H6A—C6—H6B	108.9	C19B—C20B—Zr1	75.3 (8)
C11—C7—C8	108.6 (2)	C21B—C20B—Zr1	75.0 (7)
C11—C7—C12	125.2 (3)	C25B—C20B—Zr1	122.2 (10)
C8—C7—C12	126.1 (3)	C17B—C21B—C20B	108.2 (9)
C11—C7—Zr1	74.70 (13)	C17B—C21B—C26B	125.5 (8)
C8—C7—Zr1	74.57 (13)	C20B—C21B—C26B	125.8 (8)
C12—C7—Zr1	120.79 (17)	C17B—C21B—Zr1	74.3 (7)
C7—C8—C9	108.1 (2)	C20B—C21B—Zr1	73.4 (7)
C7—C8—C13	125.5 (3)	C26B—C21B—Zr1	124.7 (10)
C9—C8—C13	126.3 (3)	C17B—C22B—H22D	109.5
C7—C8—Zr1	73.51 (13)	C17B—C22B—H22E	109.5
C9—C8—Zr1	74.99 (13)	H22D—C22B—H22E	109.5
C13—C8—Zr1	120.81 (17)	C17B—C22B—H22F	109.5
C8—C9—C10	107.8 (2)	H22D—C22B—H22F	109.5
C8—C9—C14	127.4 (3)	H22E—C22B—H22F	109.5
C10—C9—C14	124.4 (3)	C18B—C23B—H23D	109.5
C8—C9—Zr1	73.21 (12)	C18B—C23B—H23E	109.5
C10—C9—Zr1	74.23 (12)	H23D—C23B—H23E	109.5
C14—C9—Zr1	124.35 (15)	C18B—C23B—H23F	109.5
C9—C10—C11	107.4 (2)	H23D—C23B—H23F	109.5
C9—C10—C15	124.8 (3)	H23E—C23B—H23F	109.5
C11—C10—C15	127.2 (3)	C19B—C24B—H24D	109.5
C9—C10—Zr1	73.99 (12)	C19B—C24B—H24E	109.5
C11—C10—Zr1	72.98 (13)	H24D—C24B—H24E	109.5
C15—C10—Zr1	125.56 (18)	C19B—C24B—H24F	109.5
C7—C11—C10	108.1 (2)	H24D—C24B—H24F	109.5
C7—C11—C16	123.2 (3)	H24E—C24B—H24F	109.5
C10—C11—C16	127.3 (3)	C20B—C25B—H25D	109.5
C7—C11—Zr1	73.61 (13)	C20B—C25B—H25E	109.5
C10—C11—Zr1	74.99 (13)	H25D—C25B—H25E	109.5
C16—C11—Zr1	127.92 (18)	C20B—C25B—H25F	109.5
C7—C12—H12A	109.5	H25D—C25B—H25F	109.5
C7—C12—H12B	109.5	H25E—C25B—H25F	109.5
H12A—C12—H12B	109.5	C21B—C26B—H26D	109.5
C7—C12—H12C	109.5	C21B—C26B—H26E	109.5
H12A—C12—H12C	109.5	H26D—C26B—H26E	109.5
H12B—C12—H12C	109.5	C21B—C26B—H26F	109.5
C8—C13—H13A	109.5	H26D—C26B—H26F	109.5
C8—C13—H13B	109.5	H26E—C26B—H26F	109.5
H13A—C13—H13B	109.5	C28—C27—H27A	109.5
C8—C13—H13C	109.5	C28—C27—H27B	109.5
H13A—C13—H13C	109.5	H27A—C27—H27B	109.5
H13B—C13—H13C	109.5	C28—C27—H27C	109.5
C9—C14—H14A	109.5	H27A—C27—H27C	109.5
C9—C14—H14B	109.5	H27B—C27—H27C	109.5
H14A—C14—H14B	109.5	C27—C28—C29	113.4 (3)
C9—C14—H14C	109.5	C27—C28—H28A	108.9
H14A—C14—H14C	109.5	C29—C28—H28A	108.9
H14B—C14—H14C	109.5	C27—C28—H28B	108.9
C10—C15—H15A	109.5	C29—C28—H28B	108.9
C10—C15—H15B	109.5	H28A—C28—H28B	107.7
H15A—C15—H15B	109.5	C29^ii^—C29—C28	115.0 (3)
C10—C15—H15C	109.5	C29^ii^—C29—H29A	108.5
H15A—C15—H15C	109.5	C28—C29—H29A	108.5
H15B—C15—H15C	109.5	C29^ii^—C29—H29B	108.5
C11—C16—H16A	109.5	C28—C29—H29B	108.5
C11—C16—H16B	109.5	H29A—C29—H29B	107.5
H16A—C16—H16B	109.5		
			
C1—C2—C3—C6^i^	−163.3 (2)	Zr1—C19A—C20A—C21A	65.5 (2)
C4^i^—C2—C3—C6^i^	17.5 (3)	C18A—C19A—C20A—C25A	172.6 (3)
N2—C4—C5—C6	−159.1 (2)	C24A—C19A—C20A—C25A	4.0 (6)
C2^i^—C4—C5—C6	21.3 (3)	Zr1—C19A—C20A—C25A	−119.2 (3)
C4—C5—C6—C3^i^	−31.9 (3)	C18A—C19A—C20A—Zr1	−68.2 (2)
C11—C7—C8—C9	−0.1 (3)	C24A—C19A—C20A—Zr1	123.3 (4)
C12—C7—C8—C9	175.2 (2)	C19A—C20A—C21A—C17A	1.7 (3)
Zr1—C7—C8—C9	−67.62 (15)	C25A—C20A—C21A—C17A	−173.8 (3)
C11—C7—C8—C13	−176.0 (2)	Zr1—C20A—C21A—C17A	67.6 (2)
C12—C7—C8—C13	−0.7 (4)	C19A—C20A—C21A—C26A	179.3 (3)
Zr1—C7—C8—C13	116.4 (2)	C25A—C20A—C21A—C26A	3.8 (5)
C11—C7—C8—Zr1	67.57 (16)	Zr1—C20A—C21A—C26A	−114.8 (3)
C12—C7—C8—Zr1	−117.1 (2)	C19A—C20A—C21A—Zr1	−65.9 (2)
C7—C8—C9—C10	−0.1 (2)	C25A—C20A—C21A—Zr1	118.6 (3)
C13—C8—C9—C10	175.8 (2)	C18A—C17A—C21A—C20A	0.0 (3)
Zr1—C8—C9—C10	−66.71 (15)	C22A—C17A—C21A—C20A	174.2 (3)
C7—C8—C9—C14	−172.6 (2)	Zr1—C17A—C21A—C20A	−67.7 (2)
C13—C8—C9—C14	3.3 (4)	C18A—C17A—C21A—C26A	−177.6 (3)
Zr1—C8—C9—C14	120.7 (2)	C22A—C17A—C21A—C26A	−3.4 (5)
C7—C8—C9—Zr1	66.63 (16)	Zr1—C17A—C21A—C26A	114.7 (3)
C13—C8—C9—Zr1	−117.4 (2)	C18A—C17A—C21A—Zr1	67.7 (2)
C8—C9—C10—C11	0.2 (2)	C22A—C17A—C21A—Zr1	−118.1 (3)
C14—C9—C10—C11	173.0 (2)	C21B—C17B—C18B—C19B	−0.4 (16)
Zr1—C9—C10—C11	−65.84 (15)	C22B—C17B—C18B—C19B	−173.0 (14)
C8—C9—C10—C15	−171.5 (2)	Zr1—C17B—C18B—C19B	66.8 (11)
C14—C9—C10—C15	1.4 (4)	C21B—C17B—C18B—C23B	165.3 (14)
Zr1—C9—C10—C15	122.5 (2)	C22B—C17B—C18B—C23B	−7 (2)
C8—C9—C10—Zr1	66.03 (15)	Zr1—C17B—C18B—C23B	−127.5 (14)
C14—C9—C10—Zr1	−121.1 (2)	C21B—C17B—C18B—Zr1	−67.2 (9)
C8—C7—C11—C10	0.2 (3)	C22B—C17B—C18B—Zr1	120.3 (14)
C12—C7—C11—C10	−175.2 (2)	C17B—C18B—C19B—C20B	−0.9 (17)
Zr1—C7—C11—C10	67.66 (16)	C23B—C18B—C19B—C20B	−166.6 (15)
C8—C7—C11—C16	167.5 (2)	Zr1—C18B—C19B—C20B	65.6 (11)
C12—C7—C11—C16	−7.8 (4)	C17B—C18B—C19B—C24B	178.2 (17)
Zr1—C7—C11—C16	−125.0 (2)	C23B—C18B—C19B—C24B	13 (3)
C8—C7—C11—Zr1	−67.49 (16)	Zr1—C18B—C19B—C24B	−115.3 (18)
C12—C7—C11—Zr1	117.2 (2)	C17B—C18B—C19B—Zr1	−66.6 (9)
C9—C10—C11—C7	−0.2 (3)	C23B—C18B—C19B—Zr1	127.8 (15)
C15—C10—C11—C7	171.2 (2)	C18B—C19B—C20B—C21B	1.9 (17)
Zr1—C10—C11—C7	−66.74 (16)	C24B—C19B—C20B—C21B	−177.2 (16)
C9—C10—C11—C16	−166.9 (2)	Zr1—C19B—C20B—C21B	68.7 (9)
C15—C10—C11—C16	4.5 (4)	C18B—C19B—C20B—C25B	173.9 (15)
Zr1—C10—C11—C16	126.5 (3)	C24B—C19B—C20B—C25B	−5 (3)
C9—C10—C11—Zr1	66.52 (15)	Zr1—C19B—C20B—C25B	−119.3 (15)
C15—C10—C11—Zr1	−122.1 (3)	C18B—C19B—C20B—Zr1	−66.8 (11)
C21A—C17A—C18A—C19A	−1.7 (3)	C24B—C19B—C20B—Zr1	114.1 (18)
C22A—C17A—C18A—C19A	−175.9 (3)	C18B—C17B—C21B—C20B	1.6 (15)
Zr1—C17A—C18A—C19A	64.9 (2)	C22B—C17B—C21B—C20B	174.2 (13)
C21A—C17A—C18A—C23A	165.6 (3)	Zr1—C17B—C21B—C20B	−66.1 (9)
C22A—C17A—C18A—C23A	−8.6 (5)	C18B—C17B—C21B—C26B	−170.6 (13)
Zr1—C17A—C18A—C23A	−127.8 (3)	C22B—C17B—C21B—C26B	2 (2)
C21A—C17A—C18A—Zr1	−66.56 (19)	Zr1—C17B—C21B—C26B	121.8 (14)
C22A—C17A—C18A—Zr1	119.2 (3)	C18B—C17B—C21B—Zr1	67.7 (9)
C17A—C18A—C19A—C20A	2.7 (4)	C22B—C17B—C21B—Zr1	−119.7 (14)
C23A—C18A—C19A—C20A	−164.5 (3)	C19B—C20B—C21B—C17B	−2.2 (16)
Zr1—C18A—C19A—C20A	67.7 (2)	C25B—C20B—C21B—C17B	−174.0 (14)
C17A—C18A—C19A—C24A	170.6 (4)	Zr1—C20B—C21B—C17B	66.7 (9)
C23A—C18A—C19A—C24A	3.4 (6)	C19B—C20B—C21B—C26B	170.0 (14)
Zr1—C18A—C19A—C24A	−124.4 (4)	C25B—C20B—C21B—C26B	−2 (2)
C17A—C18A—C19A—Zr1	−65.0 (2)	Zr1—C20B—C21B—C26B	−121.1 (14)
C23A—C18A—C19A—Zr1	127.8 (4)	C19B—C20B—C21B—Zr1	−68.9 (11)
C18A—C19A—C20A—C21A	−2.7 (4)	C25B—C20B—C21B—Zr1	119.2 (15)
C24A—C19A—C20A—C21A	−171.3 (4)	C27—C28—C29—C29^ii^	−178.9 (4)

Symmetry codes: (i) −*x*+2, −*y*+1, −*z*+2; (ii) −*x*+2, −*y*, −*z*+2.
