# Supplementary figures and images for: Crystal structure of bis­{μ2-[(2-imino­cyclo­pentyl­idene)methyl­idene]aza­nido-κ2 N:N′}bis­[(η5-penta­methyl­cyclo­penta­dien­yl)zirconium(IV)] hexane monosolvate
Source: Acta Crystallogr E Crystallogr Commun. 2015 Nov 14;71(Pt 12):m219–20. doi: 10.1107/S2056989015021234 (PMC4719842; doi:10.1107/S2056989015021234)

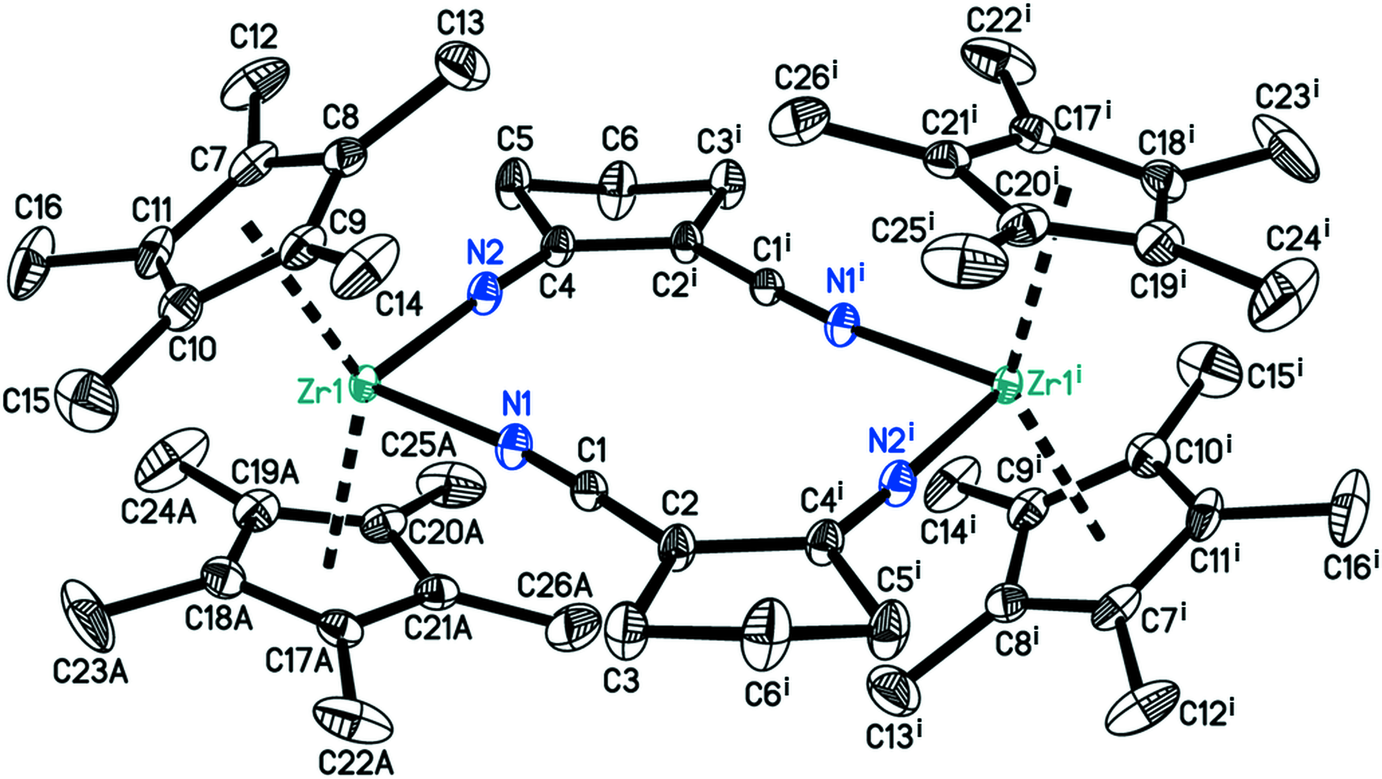

Supplement: Supplementary file 3 [file e-71-0m219-fig1.tif]

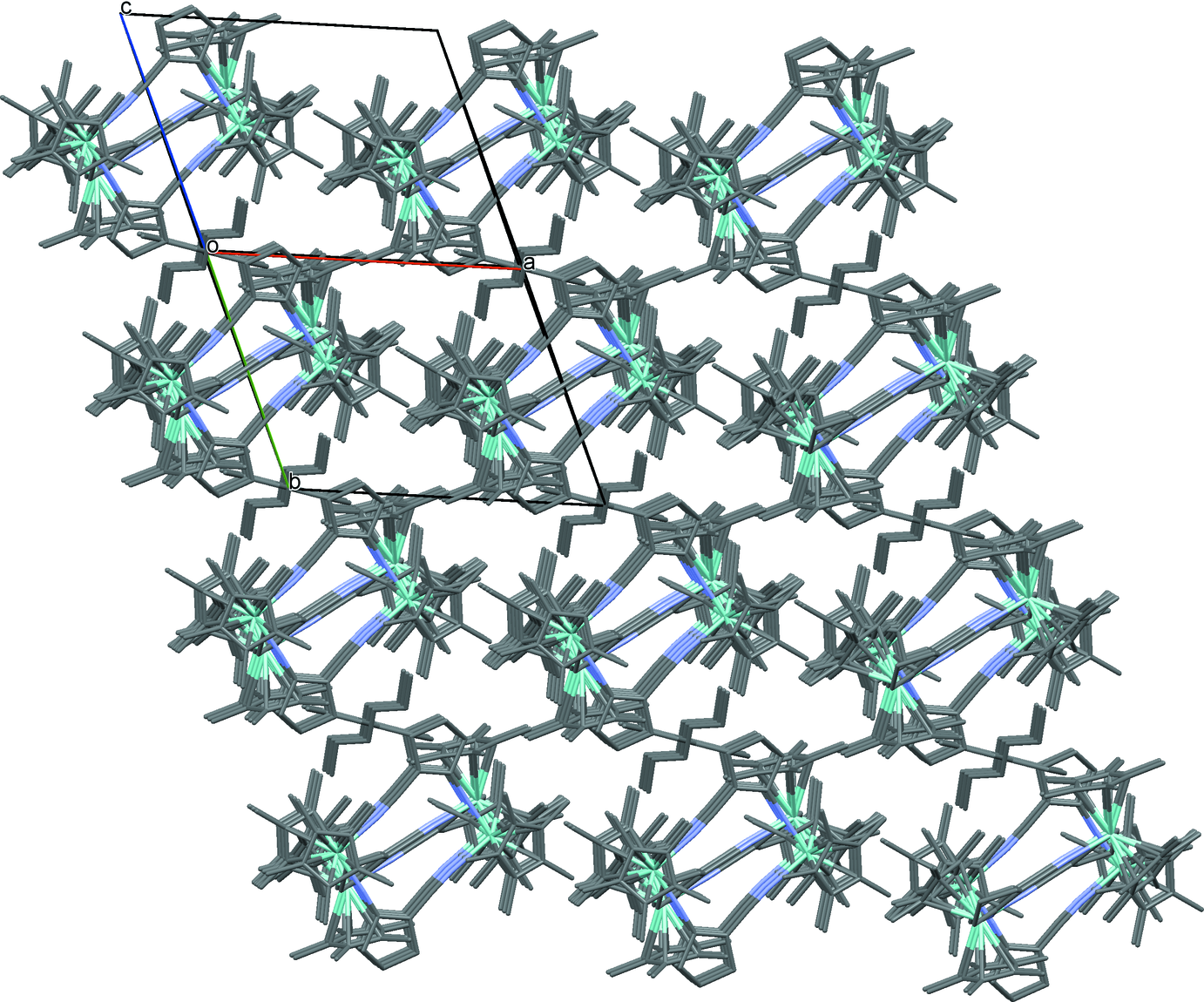

Supplement: Supplementary file 4 [file e-71-0m219-fig2.tif]
